# The Midwives Bill

**Published:** 1900-02-03

**Authors:** 


### The Hospital

A Journal of

The Medical Sciences and Hospital Administration.

Vol. XXVII.—No. 697.] Edited by Sir Henry Burdett, K.C.B., and Solomon C. Smith, M.D., M.R.C.P. [February 3, 1900.

### THE MIDWIVES BILL.

Parliament meets this year in the midst of such grave events, and is immediately confronted with questions of such serious national importance, that it may perhaps be doubted whether it will be possible to obtain for ordinary domestic legislation that attention which it would attract in quieter and more peaceful times. Nevertheless, war or no war, the great machine of national life has to go on, and if it is to go on well, even the smallest screw must be kept well oiled and the most unimportant bearing be kept bright; and this is our excuse, at such a time as the present, for turning attention afresh to the legislation which is proposed for the purpose of promoting the education and control of midwives. We have received from the Midwives Bill Committee a copy of the Bill which it is proposed to bring before Parliament this session, and as in some senses it is a good Bill, and as there are evidences that a reasonable attempt has been made to bring its clauses into some sort of accord with the conditions laid down by the General Medical Council, it deserves careful and impartial consideration.

The general aim of the Bill is to prevent any woman taking or using the name or title of midwife (either alone or in combination with any other word or words), or any name, title, addition, or description, implying that she is certified under this Act, or is qualified to act as a midwife, unless she is so certified. But it goes further than this, for it imposes its penalty not only on those who take the name of midwife, but upon any woman who "habitually and for gain" attends women in childbirth, unless she is certified under the Act. The whole machinery of the Bill is framed with the apparent intention of entirely restricting the practise of midwifery (by women other than medical practitioners) to those who have been educated and certified in accordance with its provisions. With this object a Central Midwives Board is to be created, consisting of (1) four registered medical practitioners, appointed one each by the Royal College of Physicians, the Royal College of Surgeons, the Society of Apothecaries, and the Midwives Institute; (2) two persons (one of whom shall be a woman), appointed for terms of three years by the Lord President of the Council; and (3) one person, also appointed for terms of three years by the Association of County Councils. Among the duties of this Board will be to appoint examiners; to decide upon the places where, and the times when, examinations shall be held; to publish annually a roll or general list of midwives who have been duly certified; to decide upon the removal from, or the restoration to, this roll of the name of any midwife; to issue certificates; and, subject always to the approval of the General Medical Council, to frame rules regulating the course of training, the conduct of examinations, the admission to the roll of women already in practise, and deciding the conditions under which the midwives may be suspended from practise. When in accordance with the regulations of the Central Midwives Board a woman has obtained access to the roll, she must apply to the local supervising authority (the county council or the council of county borough) of the district in which she proposes to practise, and this supervising authority will, for the fee of one shilling, issue to her a licence, which must be renewed each year, allowing her to practise within the area of the said county or county borough. Without this local licence she cannot practise even though fully certified. The supervising authority will investigate charges of malpractise, and report to the Central Board; may suspend any midwife from practise if such action should appear necessary to prevent the spread of infection; and will undertake various other duties. In regard, moreover, to all its duties, it may delegate its powers to any district council within its area, but the supervision of the midwife by the local medical officer of health, which was a prominent part of the Bill of last year, has entirely disappeared from the present one, in which there seems to be no provision for local medical control of midwives. The supervising authority is either a county, borough, or district council. A new and important clause is inserted empowering the county councils to pay any sum required from time to time to make good any deficiency in the funds of the Central Midwives Board, the various county councils contributing in proportion to the number of midwives licensed to practise in the several counties.

Now, as to the possible efficiency of such a Bill as this, everything must depend upon the completeness with which all its clauses are accepted by Parliament, and the loyalty with which its provisions are carried out. Putting on one side all the hard words which have been spoken by many medical men, and going back to the fundamental principle laid down by the General Medical Council, namely, that "it is desirable that measures should be taken to protect lying-in women of the poorer classes from the grave risk which they now incur from the unregulated practice of mid-wives," and that to this end "it is desirable that every woman who, not being a registered medical practitioner, engages for gain in practise as a midwife should be subjected to legal regulation and control," and taking this as the formal pronouncement of the highest medical authority, we cannot but admit that the Bill as it stands would go a long way towards the achievement of the end proposed. It has, however, yet to go before Parliament, and it is very doubtful whether it will run the gauntlet without suffering serious damage and defacement. It is in many ways a compromise, and some of its strongest supporters, although willing to accept the Bill rather than go without one at all, would probably not be consumed with grief if a cruel Parliament were to snip off some of its clauses. This is the danger. As it stands, with a properly constituted Central Board, and the loyal co-operation of the county councils, it ought not to be impossible to get out of the Bill the good which is sought, tainted but little with the evils which are feared. But a slight alteration in its wording, or a little laxity in its administration, might easily render it both mischievous and useless.

